# X-ray structure determination and deuteration of nattokinase

**DOI:** 10.1107/S0909049513020700

**Published:** 2013-09-25

**Authors:** Yasuhide Yanagisawa, Toshiyuki Chatake, Sawa Naito, Tadanori Ohsugi, Chieko Yatagai, Hiroyuki Sumi, Akio Kawaguchi, Kaori Chiba-Kamosida, Megumi Ogawa, Tatsumi Adachi, Yukio Morimoto

**Affiliations:** a Faculty of Pharmaceutical Sciences, Chiba Institute of Science, 15-8 Shiomi-cho, Cho-shi, Chiba 288-025, Japan; b Research Reactor Institute, Kyoto University, Asashironishi 2, Kumatori, Osaka 590-0494, Japan; c Department of Life Science, Kurashiki University of Science and the Arts, 2640 Nishinoura, Tsurajima-cho, Kurashiki, Okayama 712-8505, Japan; d Nippon Advanced Technology Co. Ltd, J-PARC, 2-4 Shirane Shirakata, Tokai, Ibaraki 319-1195, Japan

**Keywords:** nattokinase, *Bacillus subtilis natto*, deuteration, X-ray structure

## Abstract

Nattokinase (NK) is a strong fibrinolytic enzyme, which is produced in abundance by *Bacillus subtilis natto*. Although NK is a member of the subtilisin family, it displays different substrate specificity when compared with other subtilisins. The results of molecular simulations predict that hydrogen arrangements around Ser221 at the active site probably account for the substrate specificity of NK. Therefore, neutron crystallographic analysis should provide valuable information that reveals the enzymatic mechanism of NK. In this report, the X-ray structure of the non-hydrogen form of undeuterated NK was determined, and the preparation of deuterated NK was successfully achieved. The non-hydrogen NK structure was determined at 1.74 Å resolution. The three-dimensional structures of NK and subtilisin E from *Bacillus subtilis* DB104 are near identical. Deuteration of NK was carried out by cultivating *Bacillus subtilis natto* in deuterated medium. The D_2_O resistant strain of *Bacillus subtilis natto* was obtained by successive cultivation rounds, in which the concentration of D_2_O in the medium was gradually increased. NK was purified from the culture medium and its activity was confirmed by the fibrin plate method. The results lay the framework for neutron protein crystallography analysis.

## Abstract 2

X-ray structure determination and deuteration of nattokinase were performed to facilitate neutron crystallographic analysis.

## 1. Introduction

Natto is a Japanese traditional fermented food made from soybeans, and is considered a health-food product in Japan, because it is rich in proteins and valuable chemical compounds such as nattokinase (NK; Sumi *et al.*, 1987 ▶) and vitamin K2 (Sakano *et al.*, 1988 ▶). NK is produced in abundance by the fermentation of soybeans using *Bacillus subtilis natto* and extracted to medium by sporulation. NK shows strong fibrinolytic activity (Sumi *et al.*, 1992 ▶) and also activates particular fibrinolytic enzymes such as pro-urokinase (Sumi *et al.*, 1996 ▶) and tissue plasminogen activator (Yatagai *et al.*, 2008 ▶). Hence, the fibrinolytic activity derived from NK is enhanced in tissues. These previous reports indicate the potential for medical usage of NK, as in the case of therapeutic and diagnostic agent for thrombosis.

NK is a serine protease composed of 275 amino acids (molecular weight 27.7 kDa) and belongs to the subtilisin family; however, Sumi and co-workers found that the substrate specificity of NK is different from other subtilisins such as subtilisin BPN′ and subtilisin Carlsberg (Sumi *et al.*, 2011 ▶). NK has high sequence homology to subtilisin E (SE) from *Bacillus subtilis* DB104 (Wong & Doi, 1986 ▶). The difference between the two sequences is only Ala192 (SE) → Val192 (NK). Previously, the X-ray structure of a mutant SE (mut-SE) was determined at 2.0 Å resolution (Jain *et al.*, 1998 ▶). The substitution Ser221 → Cys221 was introduced to block the activity site of mut-SE; hence a precursor molecule including 77 additional amino acids was bound at the N-terminus of mut-SE. The three-dimensional structure of NK was predicted based on the SE structure (Zheng *et al.*, 2004 ▶, 2006 ▶), suggesting that the specific function of NK would be closely related to the hydroxyl-rich environment around Ser221 at the active site. However, the three-dimensional structure of the mature SE has not been revealed. In addition, H atoms could not be determined by previous X-ray analysis because of the medium resolution of the X-ray data. The aim of our study was to reveal the reaction mechanism of NK based on the precise arrangement of H atoms in the NK structure using neutron protein crystallography.

Here, the X-ray crystallographic analysis of the non-hydrogen structure of NK is presented and the production of deuterated NK from *Bacillus subtilis natto* was performed, thereby providing the framework to solve the hydrogen arrangements of NK. X-ray structure determination is essential for neutron protein crystallography, because the non-hydrogen structure is necessary to determine the initial phases of the neutron diffractions by the molecular replacement method, and the deuteration of NK is expected to improve the quality of neutron diffraction data of NK.

Deuteration of biomacromolecules is an experimental technique to replace H atoms of biomacromolecules with deuterium (D) atoms. The intensity of neutron scattering from a D atom (45.0 fm^2^) is more than three-fold higher than an H atom (14.0 fm^2^). Moreover, incoherent scattering, which causes background noise in neutron measurements, of a D atom (2.0 barn) is much smaller than the incoherent scattering of an H atom (79.9 barn). Therefore, prior to almost every neutron diffraction experiment, targeted biomacromolecules should be deuterated. The simplest approach for deuteration in neutron crystallographic studies is to use heavy water (D_2_O) as the solvent instead of water (H_2_O). H atoms of H_2_O and exchangeable H atoms of functional groups such as amino and hydroxyl groups are exchanged with D atoms in this method; however, H atoms of C—H bonds cannot be exchanged. In order to obtain higher-quality neutron data, biomacromolecules have to be obtained from bacteria grown in deuterated medium. Although both water and chemical reagents of the culture medium must be deuterated for complete deuteration, using only deuterated solvent (hereafter D_2_O culture medium) in the culture can achieve substantially deuteration of the target biomacromolecule. In the present study, we obtained a D_2_O resistant strain of *Bacillus subtilis natto* by successive cultivation, and deuterated NK was purified from the culture medium. The enzymatic activity of the deuterated NK was assessed by the fibrin plate method (Astrup & Mullertz, 1952 ▶).

## 2. Materials and methods

### 2.1. X-ray structure determination

Purification, crystallization and X-ray data collection were described in our previous publication (Yanagisawa *et al.*, 2010 ▶). Native nattokinase powder was provided by Honda Trading CC and crystallized using the sitting-drop vapour-diffusion method after gel-filtration chromatography. Needle-like crystals could be obtained from a solution containing 6.4 mg ml^−1^ NK, 50 m*M* HEPES (pH 7.5), 10% polyethylene glycol 8000 and 8% ethylene glycol. X-ray diffraction data were collected using synchrotron radiation at SPring-8, Japan. The initial phases were determined by the molecular replacement method using the atomic coordinates of amino-acid residues 1–175 of SE (PDB entry 1scj; Jain *et al.*, 1998 ▶). The atomic models were rebuilt and refined using *COOT* (Emsley *et al.*, 2010 ▶) and *PHENIX* (Adams *et al.*, 2010 ▶). *R*-factor and *R*_free_ of the final structure were 0.133 and 0.199, respectively. Statistics of the structure determination are summarized in Table 1 ▶.

### 2.2. Deuteration of nattokinase

*Bacillus subtilis natto* Miyagino (BSNM) was used as the starter culture. Initially, 300 ml of 2% polypeptone S and 3% glycerol were placed in a 500 ml Erlenmeyer flask and sterilized by heating at 393 K for 20 min. Medium containing a loopful of BSNM, 2% polypeptone S and 3% glycerol BSNM was prepared for pre-cultivation. The medium was incubated at 310 K for 2 d in a shaking incubator (100 r.p.m.). Two microliters of the pre-cultured BSNM medium was transferred into 5 ml liquid medium, and incubated at 310 K with shaking (1200 r.p.m.). After 7 or 14 days cultivation, the fibrinolytic activity of NK extracted from BSNM was assessed using the fibrin plate method (Astrup & Mullertz, 1952 ▶). Thirty microliters of cultured medium was applied onto a fibrin plate in a petri dish (diameter = 8.5 cm) and the fibrinolysis area was measured after 1 h and 4 h. The BSNM medium, which has the highest activity of NK in the assessment, was used for the next starter culture. The concentration of D_2_O was gradually increased in successive cultivations. Each cultivation step was carried out in duplicate in test tubes, and the strongest growing culture, which had highest activity of NK, was used in the next cultivation round. When the activity of NK of the BSNM had degraded, the culture was retried at the same concentration of D_2_O using the same or the former generation of BSNM. BSNM that grew in 100% deuterated medium and produced sufficiently good yields of NK was obtained after 7 and 8 passage cultivation steps.

In the present study, BSNM was cultured in an open system; therefore, H_2_O contamination during cultivation was assessed by a neutron transmission experiment. Neutron transmission was measured using 4cnd (Shibuya *et al.*, 1968 ▶) at the Research Reactor of Kyoto University. 0, 50 and 100% deuterated culture medium before and after cultivation were used for the neutron experiment. BSNM and solid matter were removed from the medium by centrifugation and filtration before sealing the medium into a capillary (diameter = 4 mm). The capillary was mounted on an aluminium mounter and a monochromatic neutron beam (λ = 1.0 Å) was collimated at the sample position by cadmium sheets. Each measurement was carried out for 10 min at 5 MW. The exposure time was adjusted using the neutron monitor, which was located between the monochromator and the goniometer.

Deuterated and non-deuterated NK were purified from the cultured medium of BSNM. The 11 ml supernatants of 45 ml cultured medium of 0 and 100% D_2_O were concentrated to 1 and 1.5 ml, respectively. The two types of NK solution were purified by gel-filtration chromatography and hydrophobic interaction chromatography. Partial purification was initially carried out by gel filtration on a Sephadex G10 column. The second purification step involved hydrophobic interaction chromatography using a butyl-Sepharose FF column. A gradient from 2 to 0 *M* AS was used, with a flow rate of 1.0 ml min^−1^ and a total elution period of 50 min. The protein was further purified by gel filtration using a Sephacryl-S100 column and the resulting solution was concentrated to 45 µl and 42 µl using centrifugal filter units.

## 3. Results and discussion

### 3.1. X-ray structure of NK

**Figure 1:**
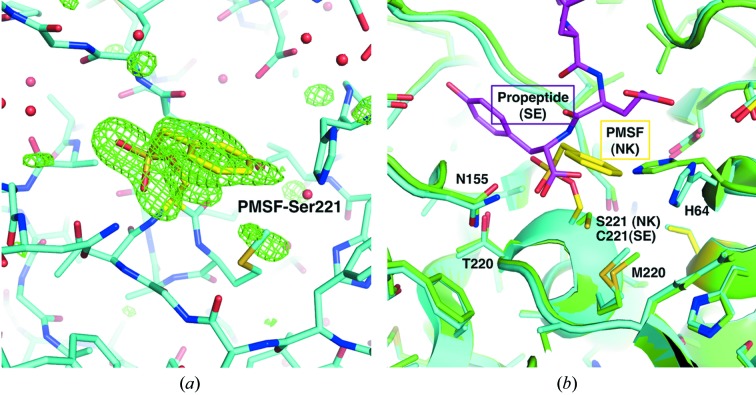
Stick model around Ser221 located at the active site of NK. (*a*) Stick model of NK and PMSF covalently bound to Ser221. C atoms of NK and PMSF are coloured cyan and yellow, respectively. The |*F*_o_| − |*F*_c_| map omitting Ser221 and PMSF at the 2σ level has been superimposed onto the model. (*b*) Combinations of the cartoon model (main chain) and stick model (side chain) of NK and SE. The SE structure was superimposed onto the NK structure using residues 1–275. Cartoon models of NK and SE are coloured cyan and green, respectively. The C atoms of a propeptide, which is complexed with SE, are coloured magenta.

All 275 residues of NK could be determined in the present X-ray analysis. Previous mass spectroscopic analysis indicated the possibility of glycosylation or other chemical reactions (Chiba-Kamoshida *et al.*, 2010 ▶); however, no modification was observed, except Ser221 at the active site. Phenylmethylsulfonyl fluoride (PMSF) was covalently bound to the hydroxyl group of Ser221 (Fig. 1*a* ▶), because PMSF was present in the buffer during the purification process in order to prevent self-digestion. This result suggests that serine protease inhibitors, like PMSF, should not be used in our neutron crystallography analysis. The averaged r.m.s.d. values for the main chain and side chains between NK and SE were 0.25 and 0.45 Å, respectively. Structural differences were mainly observed for regions of SE interacting with the bound propeptide, and the substitution Ala192 (SE) → Val192 (NK) did not seem to affect directly the active site of these enzymes (Fig. 1*b* ▶). Therefore, the prediction that the specific function of NK is closely linked to the hydroxyl-rich environment around Ser221 at the active site (Zheng *et al.*, 2004 ▶, 2006 ▶), which was proposed by molecular simulations using the SE structure as the starting model, would be plausible.

The substrate specificity of NK is different from subtilisins BPN′ and Carlsberg (Sumi *et al.*, 2011 ▶); although the r.m.s.d. values for the main chain from subtilisin BPN′ (PDB ID 1sbn; Heinz *et al.*, 1991 ▶) and subtilisin Carlsberg (PDB ID 1sel; Syed *et al.*, 1993 ▶) are 0.49 and 0.36 Å, respectively. Enzymatic activity of NK against Bz-Ile-Glu-(OR)-Gly-Arg-pNA (0.68 µmol min^−1^ on average) was higher than against the substrate Suc-Ala-Ala-Pro-Phe-pNA (0.34 µmol min^−1^). In contrast, the substrate specificities towards the two substrates are reversed in subtilisin Carlsberg and other proteases such as nagarase and orientase. At first glance, the active site of NK in the non-hydrogen structure closely resembles subtilisins BPN′ and Carlsberg, implying the importance of obtaining structural information of protonations, hydrogen-bonding networks, and hydrophobic interactions of NK to fully reveal the specific enzymatic reaction of this enzyme.

### 3.2. Deuteration of NK

The first cultivation was carried out using native BSNM in 0, 25, 50, 75 and 100% D_2_O culture medium. Strong, medium and weak NK activity were observed in 0, 25 and 50% D_2_O 14-day cultured medium, respectively, and no activity was measured in 50, 75 and 100% D_2_O medium. Therefore, the D_2_O concentration was increased in steps of 25%. While a considerable number of BSNM cultures lost NK activity during the successive cultivation rounds, a lot did maintain NK activity up to 100% D_2_O medium. The D_2_O concentration for this lot reached 100% in six successive cultivations and the final BSNM optimized for deuteration (BSNM–D_2_O) could be obtained after a further two cultivations in 100% D_2_O medium. The results from SDS electrophoresis showed that NK was produced in abundance in this bacterial culture.

Since the neutron transmission of hydrogen and deuterium atoms differ, the ratio H_2_O:D_2_O in each medium can be estimated from neutron transmission data. As shown in Table 2 ▶, the neutron transmission of the medium did not change significantly, suggesting the contamination of H_2_O during cultivation was negligible.

**Figure 2:**
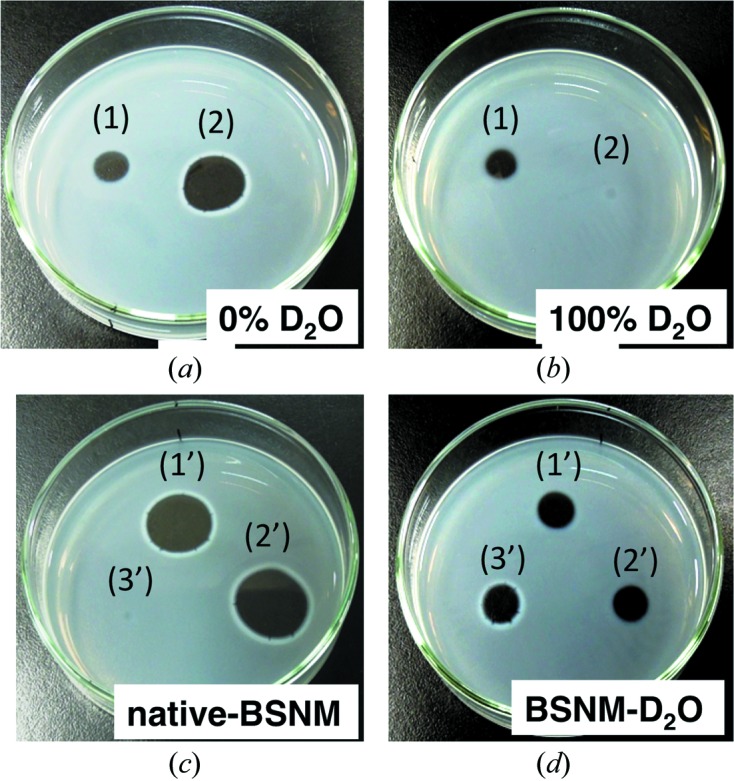
Fibrinolysis activity of native BSNM and BSNM–D_2_O grown in 0, 25, 50, 75 and 100% D_2_O medium. 30 µl of supernatant was applied onto a fibrin plate. Photographs were taken after incubation for 4 h at 310 K. (*a*) Native BSNM and BSNM–D_2_O grown in 0% D_2_O medium, (*b*) in 100% D_2_O medium. Labels 1 and 2 indicate BSNM–D_2_O and native NSBM, respectively. (*c*) Native NSBM grown in 25%–75% D_2_O medium. (*d*) BSNM–D_2_O. Labels 1′, 2′ and 3′ indicate 25%, 50%, 75% D_2_O medium, respectively.

**Figure 3:**
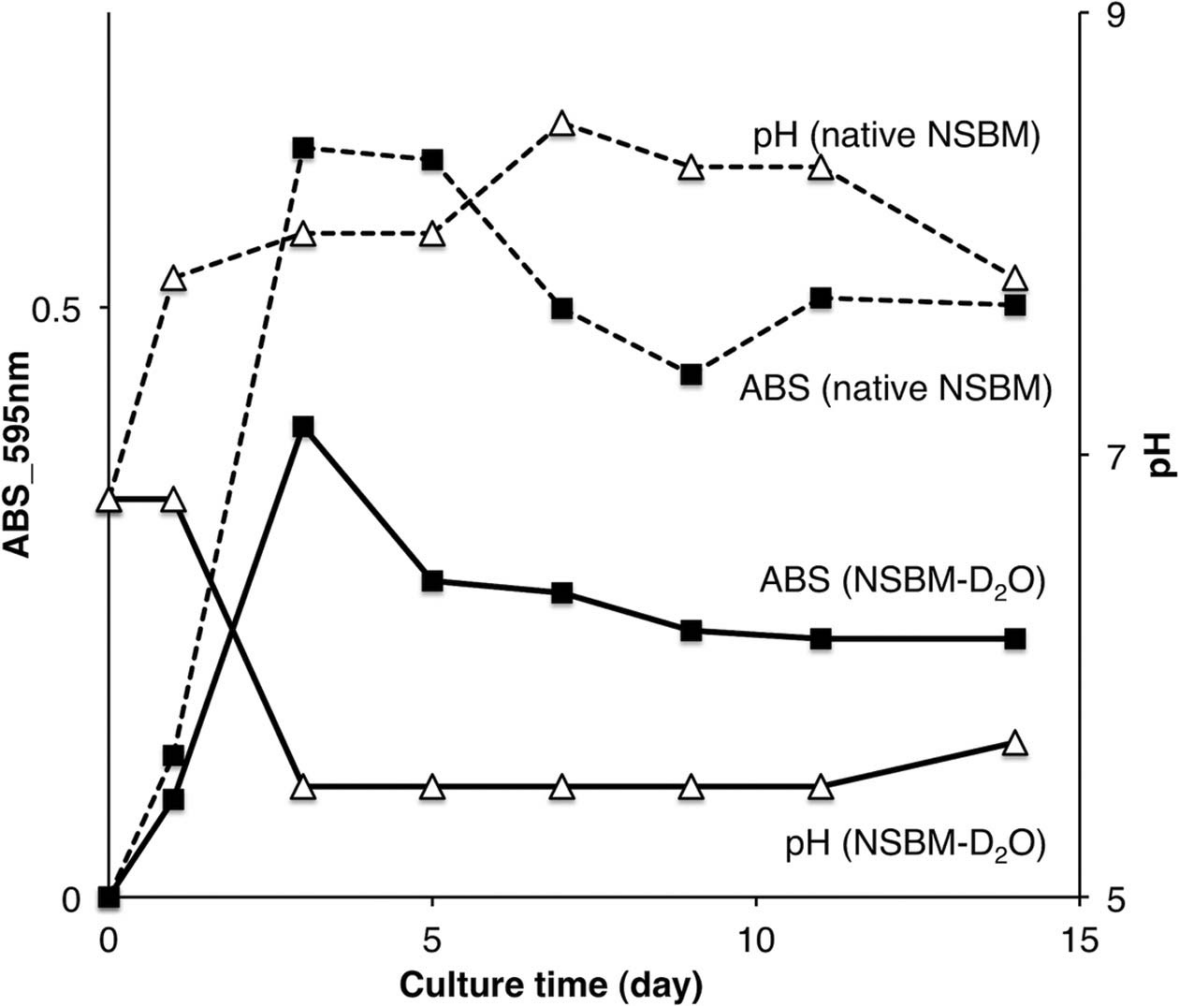
Turbidity and pH of BSNM–D_2_O in 100% D_2_O medium (solid lines) and native BSNM in 100% H_2_O medium (broken lines). Squares and triangles indicate turbidity and pH of the mediums, respectively.

Fig. 2 ▶ shows the results of the fibrin plate test; the fibrinolysis area corresponds to NK activity, of native BSNM and BSNM–D_2_O grown in 0, 25, 50, 75 and 100% D_2_O medium. NK activity of native BSNM was very strong in 0% D_2_O medium; however, it decreased with increasing D_2_O concentration, and could not be observed in 75% and 100% medium. On the other hand, the NK activity of BSNM–D_2_O was almost the same at each D_2_O percentage. While the results indicated that D_2_O strongly inhibited growth and NK production of the native BSNM, BSNM–D_2_O had acquired resistance to D_2_O. The reason why the NK activity of BSNM–D_2_O was constant between 0 and 100% D_2_O concentrations can be explained by the growth curve and pH of their medium. In general, the growth curve of BSNM fluctuates every three to five days; BSNM repeats proliferation and sporulation during cultivation. However, BSNM–D_2_O underwent sporulation only once on the third day in a 14-day period (Fig. 3 ▶). On the third day, the pH of the medium suddenly dropped from a neutral (6.8) to an acidic (5.5) value and never recovered. The lower pH prevented the next proliferation; only an amount of NK production, which one sporulation made, could be obtained at various D_2_O concentrations. The control of the pH appears to be necessary to obtain more deuterated NK using BSNM–D_2_O. 1.6 µg of purified NK could be obtained from 45 ml 100% D_2_O cultured medium of BSNM–D_2_O, and the NK activity was confirmed by the fibrin plate method using NK from those of a 100% H_2_O cultured medium of native BSNM (control).

## 4. Conclusion

In the present study, we have carried out X-ray structure determination and deuteration of NK to facilitate future neutron crystallography analysis. The X-ray structure of NK was found to be very similar to that of subtilisin E from *Bacillus subtilis*, suggesting that the hypothesis based on subtilisin E, *i.e.* that the structural location of the H atoms around Ser221 within the active site is important for enzymatic specificity, is reasonable. Therefore, neutron crystallography is expected to provide useful information for understanding the enzymatic mechanism of NK. For the first time, a D_2_O-resistant strain of *Bacillus subtilis natto* was obtained by eight successive cultivation rounds. This strain was resistant to D_2_O but lost the pH regulating system. In order to perform large-scale production of deuterated NK, the control of the pH of the medium is necessary. Purified NK could be obtained from a small-scale deuterated culture medium and its activity was confirmed.

## Supplementary Material

PDB reference: 4dww

## Figures and Tables

**Table 1:** Statistics of the X-ray experiment of NK Values indicated in parentheses represent the highest-resolution shell.

Crystallographic parameters	
Space group	*C*2
Cell dimensions (Å, °)	*a* = 74.3, *b* = 49.9, *c* = 56.3, *β* = 95.2

Data collection	
*d*_min_ (Å)	1.74
*R*_merge_	0.052 (0.153)
Completeness	0.693 (0.300)

Structure determination	
Resolution (Å)	41.4–1.74
*R*-factor	0.133 (0.143)
*R*_free_	0.199 (0.236)
R.m.s.d. bond (Å)	0.006
R.m.s.d. angle (Å)	0.974
PDB ID	4dww

**Table 2:** Neutron transmission of culture medium of BSNM–D_2_O

Culture medium	0% D_2_O	50% D_2_O	100% D_2_O
Neutron transmission			
Before cultivation	61.0	74.9	88.4
After cultivation	61.7	74.2	90.4
Pure D_2_O	62.6		
Pure H_2_O	92.7		
